# How Assisted Living Staff Conceive of Dementia Care

**DOI:** 10.1093/geroni/igaa057.2351

**Published:** 2020-12-16

**Authors:** Debra Dobbs, Sheryl Zimmerman, Stephanie Miller, Paula Carder, Anna Beeber, Jennifer Hodgkinson, Julia Thorp

**Affiliations:** 1 University of South Florida, Tampa, Florida, United States; 2 University of North Carolina at Chapel Hill, Chapel Hill, North Carolina, United States; 3 Cecil G. Sheps Center for Health Services Research, Chapel Hill, North Carolina, United States; 4 Portland State University, Portland, Oregon, United States

## Abstract

For those who provide care to the more than 40% of persons with dementia in assisted living (AL) communities, behavioral expressions (BEs) can be challenging. The objective of this mixed-methods study was to understand how AL staff conceive of BEs and what strategies they use to address them. Staff from 250 AL communities in seven states were asked to describe one successful and unsuccessful case of care. A conceptual model related to antecedents, behaviors, and consequences was developed and expanded to include staff strategies and outcomes of care; organizational characteristics associated with care practices were examined. Anxiety/restlessness, combativeness and resistance to care were the most prevalent BEs. Medical interventions (e.g., inpatient psychiatric assessment, medication management) were used in two-thirds of cases. Person-centered care was used more often in successful cases. Respondents in dementia-only communities identified antecedents to BEs more often than those in other communities.

